# Nivolumab Enhances *In Vitro* Effector Functions of PD-1^+^ T-Lymphocytes and *Leishmania*-Infected Human Myeloid Cells in a Host Cell-Dependent Manner

**DOI:** 10.3389/fimmu.2017.01880

**Published:** 2017-12-22

**Authors:** Christodoulos Filippis, Katharina Arens, Gaetan Aime Noubissi Nzeteu, Gabriele Reichmann, Zoe Waibler, Peter Crauwels, Ger van Zandbergen

**Affiliations:** ^1^Division of Immunology, Paul-Ehrlich-Institut, Langen, Germany; ^2^Immunology, Johannes Gutenberg University of Mainz, Mainz, Germany

**Keywords:** *Leishmania*, programmed death-1, programmed death-1 ligand 1, programmed death-1 ligand 2, nivolumab, human macrophages, human dendritic cells, T-cells

## Abstract

Functional impairment of T-cells and a concomitant augmented expression of programmed death-1 (PD-1) have been observed in visceral leishmaniasis patients, as well as in experimental models for visceral and cutaneous leishmaniasis. The PD-1/PD-1-ligand (PD-1/PD-L) interaction negatively regulates T-cell effector functions, which are required for parasite control during leishmaniasis. The aim of this study was to elucidate the impact of the PD-1/PD-L axis in a human primary *in vitro* infection model of *Leishmania major* (*Lm*). Blocking the PD-1/PD-L interaction with nivolumab increased T-cell proliferation and release of the proinflammatory cytokines TNFα and IFNγ during the cocultivation of *Lm-*infected human monocyte-derived macrophages (hMDMs) or dendritic cells (hMDDC) with autologous PD-1^+^-lymphocytes. As a consequence *Lm* infection decreased, being the most pronounced in hMDDC, compared to proinflammatory hMDM1 and anti-inflammatory hMDM2. Focusing on hMDDC, we could partially reverse effects mediated by PD-1 blockade by neutralizing TNFα but not by neutralizing IFNγ. Furthermore, PD-1 blockade increased intracellular expression of perforin, granulysin, and granzymes in proliferating CD4^+^-T-cells, which might be implicated in reduction of *Lm*-infected cells. In all, our data describe an important role for the PD-1/PD-L axis upon *Lm* infection using a human primary cell system. These data contribute to a better understanding of the PD-1-induced T-cell impairment during disease and its influence on immune effector mechanisms to combat *Lm* infection.

## Introduction

The parasitic disease leishmaniasis is still endemic in 97 countries, causing up to 30,000 deaths annually, a number potentially increasing due to climate changes and global warming (1). A prerequisite for controlling *Leishmania* infection is a strong adaptive immune response. Based on experimental mouse models, it is widely accepted that disease susceptibility is associated with IL-10 and IL-4 producing T-helper 2 (T_H_2) cells, whereas a strong T-helper 1(T_H_1)-mediated IFNγ production promotes healing by inducing leishmanicidal nitric oxide in the *Leishmania*-harboring cells (2). In human leishmaniasis this T_H_1/T_H_2 dichotomy does not always hold true and the resulting T-cell response strongly depends on the *Leishmania* strain and the immune status of the host (3–6). In addition, *in vitro* data from cutaneous Leishmaniasis patients show parasite control to be mediated rather by IFNγ-induced reactive oxygen species (ROS) then by nitric oxide (7, 8). Macrophages and dendritic cells, the final host cells of *Leishmania* parasites, play an important role in the initiation of the adaptive immune response. Several *in vitro* studies demonstrated *Leishmania*-naive healthy human donors to possess a natural T-cell response against live parasites, antigen extracts or specific components of different *Leishmania* strains (9–16). This early MHC class II dependent T-cell response was shown to dampen *Leishmania* parasite burden in autologous human macrophage/T-cell cocultures (11). The activation of CD8^+^- and CD4^+^-T-cells is regulated by various signals such as costimulatory molecules, which can either positively or negatively influence T-cell priming.

The coinhibitory receptor programmed death-1 (PD-1, CD279), which is a member of the B7-CD28 family, is expressed on activated T-cells and B-cells. Upon association with its ligands PD-L1 (CD274) or PD-L2 (CD273), which are expressed on, e.g., macrophages and dendritic cells, T-cell activation is suppressed by inhibition of CD28 signaling (17). The role of the PD-1/PD-L axis in T-cell exhaustion, a functional impairment of T-cells, is very well studied in the field of cancer and in chronic infections such as HIV, HCV, or lymphocytic choriomeningitis virus (LCMV) (18–20). Recent publications indicate that the PD-1/PD-L pathway may play a similar role in *Leishmania* infection (21–24). In the canine and mouse model of visceral leishmaniasis, PD-1/PD-L-mediated T-cell exhaustion together with an impaired phagocyte function was observed. Blocking the PD-1/PD-L interaction in these models partially rescued effector functions of exhausted T-cells, which resulted in a lower parasite burden (21, 23). In splenic aspirates of visceral leishmaniasis patients an anergic/exhausted CD8^+^ T-cell phenotype plus an augmented expression of PD-1 was found (24). Nevertheless, functional data regarding the involvement of the PD-1/PD-L axis in human leishmaniasis is scarce.

In this study, we aimed to define a role for the PD-1/PD-L axis during *Leishmania* infection of human primary myeloid and lymphoid cells. By using a newly established autologous *in vitro* model consisting of functionally impaired PD-1^+^-T-lymphocytes, three potential *Leishmania major* (*Lm*) host cell types and the cancer therapeutic anti-PD-1 antibody nivolumab, we could demonstrate that PD-1 blockade reinvigorated T-cell effector functions. Depending on the type of parasitized human primary myeloid cell, the magnitude of T-cell-mediated parasite elimination varied. Focusing on dendritic cells, we found PD-1 blockade-mediated effects to be partly TNFα dependent. Furthermore, PD-1 blockade enhanced almost exclusively *Lm*-induced proliferation of CD4^+^ and not CD8^+^ T-cells. Moreover, an increased expression of cytolytic T-cell effector molecules was detected, which are likely to be implicated in reduced parasite survival.

In all, our study gives insight into the role of the PD-1/PD-L axis during *Leishmania* infection of primary human cells. This information may be useful for the development of immunotherapeutic strategies targeting leishmaniasis.

## Materials and Methods

### *Lm* *Parasites*

*Leishmania major* (MHOM/IL/81/FEBNI) wild-type and transgenic parasites (dsRED) were cultured as described (11, 25, 26).

### Human Peripheral Blood Mononuclear Cells (PBMCs)

Human PBMCs were isolated from buffy coats (DRK-Blutspendedienst Hessen GmbH, 506838) from blood donations by healthy German adults without known exposure to *Leishmania* parasites. PBMC isolation was performed as described previously (11). Up to 96–99% pure monocytes (Impurities: 1–4% lymphocytes) were isolated by CD14^+^ MACS selection (Miltenyi, 130-050-201). By the use of different cytokines, monocytes were differentiated in complete medium (CM; RPMI1640, 10% FCS, 2 mM l-glutamine, 50 µM β-mercaptoethanol, 100 U/mL penicillin, 100 µg/mL streptomycin, 1 mM HEPES) into proinflammatory human monocyte-derived macrophages type 1 (hMDM1) (10 ng/mL human GM-CSF; Leukine^®^, sargramostim, Bayer HealthCare), anti-inflammatory human monocyte-derived macrophages type 2 (hMDM2) (30 ng/mL human M-CSF; R&D Systems), or human monocyte-derived dendritic cells (hMDDC) (5 ng/mL GM-CSF; 10 ng/mL human IL-4, Gibco^®^, PHC0045) for a period of 5 days at 37°C, 5% CO_2_ as described (27). CD14^−^ cells or peripheral blood lymphocytes (PBLs), respectively, were seeded in six-well plates (1 × 10^6^ cells/mL) and stimulated with 0.5 µg/mL phytohemagglutinin (PHA) (Oxoid, R30852801) in CM for 6 days.

### Infection of Human Primary Macrophages or Dendritic Cells

Human monocyte-derived macrophages or dendritic cells were detached, counted and seeded in 1.5 or 2 mL microcentrifuge tubes. For infection, stationary-phase *Lm* promastigotes (wild-type or dsRED parasites) were added at a multiplicity of infection (MOI) of 10. After 24 h of incubation at 37°C, 5% CO_2_, extracellular parasites were removed by centrifugation of microcentrifuge tubes and washing steps with CM. (Non-) infected hMDM/hMDDC were analyzed by flow cytometry or used in the CFSE-based proliferation assays (see below).

### CFSE-Based Proliferation Assay

The hMDM or hMDDC, which still contain 1–4% lymphocytes, were stained prior to *Lm* infection, using 5(6)-Carboxyfluorescein diacetate N-succinimidyl ester (CFSE) (Sigma, C1157) as described previously (11). PHA-stimulated autologous PBLs (PBLs^PHA^) were labeled with CFSE and coincubated with the (non-) infected CFSE-labeled hMDM/hMDDC, at a ratio of 5:1. The anti-PD-1 fully human IgG4 (nivolumab, Opdivo^®^, Bristol-Myers Squibb) was used for PD-1 blocking experiments at a final concentration of 0.625 µg/mL. For neutralization of cytokines 20 µg/mL anti-IFNγ (clone B27, Biolegend^®^), 20 µg/mL anti-TNFα (infliximab, Remsima^®^, Mundipharma) or 20 µg/mL Isotype Control (MOPC-21, Biolegend^®^, data not shown) were used. After 5 days of coculture at 37°C, 5% CO_2_, supernatants were frozen at −80°C and cells were collected. After immunostaining, proliferation (CFSE^low^-T-cells) and infection (*Lm* dsRED^+^ hMDM or hMDDC) were analyzed by flow cytometry. CD4^+^ and CD8^+^ PBLs^PHA^ were separated from autologous PBLs^PHA^ using CD4^+^ and CD8^+^ MACS Isolation (130-045-101, 130-045-201, Miltenyi) before the cocultivation step.

### Flow Cytometry

For flow cytometric analysis of human primary cells, at least 0.3 × 10^6^ cells were labeled with fluorescently labeled antibodies (Table S1 in Supplementary Material) and corresponding isotype controls as defined by the manufacturer. Intracellular proteins were labeled by prior fixation (4% paraformaldehyde) and permeabilization (0.5% saponin) of cells. Intranuclear transcription factors Tbet and GATA3 were labeled by using eBioscience™ Foxp3/Transcription Factor Staining Buffer Set. To label specifically nivolumab, a goat antihuman IgG-Fc polyclonal Fab_2_ R-PE (Dianova; 109-116-098) was used. Upon analyzing, at least 10,000 events (human cells) were recorded using a BD LSR II flow cytometer (BD Bioscience, Heidelberg). Data were analyzed by FlowJo software (Treestar).

### ELISA

TNFα and IFNγ levels were analyzed in the supernatants by using Human TNF-alpha DuoSet ELISA (DY008) or Human IFN-gamma DuoSet ELISA (DY285) from R&D systems according to the manufacturer’s protocol plus a TECAN^®^ Infinite F50^®^ microplate reader.

### Statistical Analysis

Samples were tested for normal Gaussian distribution using D’Agostino-Pearson omnibus normality test. In case of normally distributed paired samples a parametric paired *t*-test was performed. Otherwise, the Wilcoxon signed-rank test was used. All calculations were done using Graph-Pad Prism version 7. A value of *P* < 0.05 was considered statistically significant. Pearson’s *r* correlations were also calculated by Graph-Pad Prism version 7. *r* ≥ 0.7 indicates a positive correlation; *r* ≤ −0.7 indicates an inverse correlation.

## Results

### PD-1 Ligand Cell Surface Expression on Macrophages and Dendritic Cells Is Differently Modulated by *Leishmania* Infection

**Figure 1:**
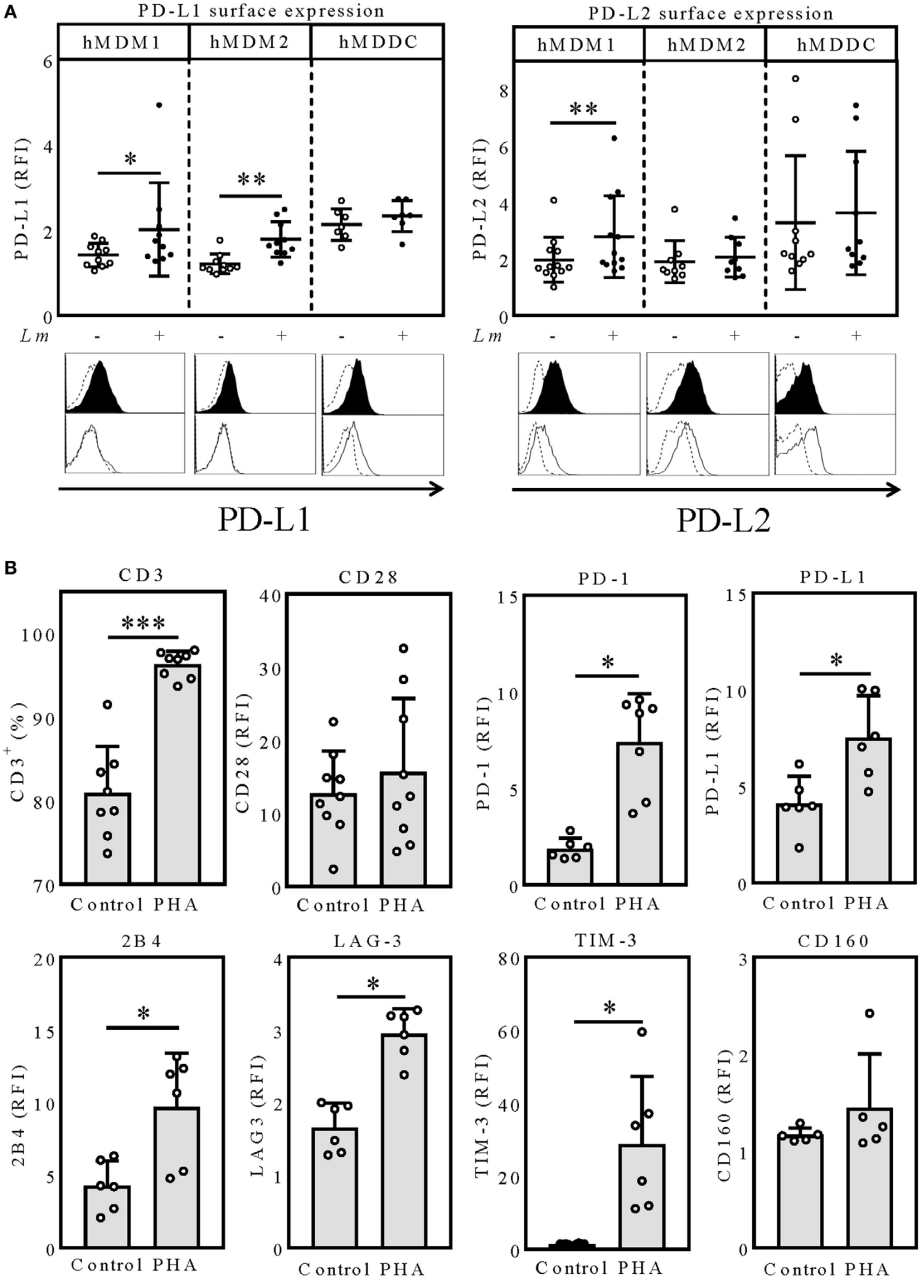
PD-L1/PD-L2 surface expression on hMDM and hMDDC after *Lm* infection. **(A)** hMDM1, hMDM2, or hMDDC were incubated with/without *Lm* (MOI 10) for 24 h and expression levels of PD-L1 or PD-L2 were determined by flow cytometry, respectively. hMDM and hMDDCs were gated using their FSC/SSC properties. Data are presented as mean ± SD RFI (ratio of the mean fluorescence intensity of specific markers to the mean fluorescence intensity of isotype controls). Representative histograms for a surface marker staining of PD-L1 and PD-L2 on infected (black filled histogram) or non-infected (black non-filled histogram) hMDMs and hMDDCs, compared to the respective isotype (black dashed line) control. Statistics were calculated by Wilcoxon matched-pairs signed rank test, *P* < 0.05 is considered statistically significant (*P < 0.05; **P < 0.01). At least three independent experiments were performed (*n* = 7–9). Expression of exhaustion marker after PHA-stimulation of PBLs. **(B)** PBLs were incubated for 6 days with/without PHA (0.5 µg/mL) and expression levels of the indicated surface molecules were determined *via* flow cytometry. T-cells were gated *via* their FSC/SSC properties and CD3. Data are presented as RFI (ratio of the mean fluorescence intensity of specific markers to the mean fluorescence intensity of isotype controls) or percentage of CD3^+^ cells. Statistics were calculated using the parametric paired *t*-test (CD3, CD28) or Wilcoxon matched-pairs signed rank test; *P* < 0.05 is considered statistically significant (*P < 0.05; **P < 0.01). At least three independent experiments were performed (*N* = 6–9). hMDM1, proinflammatory human monocyte-derived macrophages type 1; hMDM2, anti-inflammatory human monocyte-derived macrophages type 2; hMDDC, human monocyte-derived dendritic cells; *Lm, Leishmania major*; PD-L1, programmed death-1 ligand 1; PD-L2, programmed death-1 ligand 2; MFI, mean fluorescence intensity; PHA, phytohemagglutinin; PBL, peripheral blood lymphocytes; LAG-3, lymphocyte-activation gene 3; TIM-3, T-cell immunoglobulin and mucin-domain containing-3.

To assess whether PD-1/PD-Ligand (PD-1/PD-L) interactions can influence *Lm* infection of human myeloid cells, we first determined cell surface expression of PD-L1 and PD-L2 after *Lm* infection. For this purpose, we generated CD14^+^CD163^−^ proinflammatory macrophages (hMDM1), CD14^+^CD163^+^ anti-inflammatory macrophages (hMDM2), or CD1a^+^CD14^−^ dendritic cells (hMDDC) as described previously (27). We analyzed all three host cell types, as their individual roles in human *Lm* disease are insufficiently clarified. The cell surface density of both PD-L1 and PD-L2 was quantified by flow cytometry. Therefore, the relative fluorescence intensity (RFI) was measured as the ratio of the mean fluorescence intensity of specific markers to the mean fluorescence intensity of isotype controls. Low levels of PD-L1 were detected on hMDM1 (RFI: 1.42 ± 0.28) and hMDM2 (RFI: 1.21 ± 0.23). After *Lm* infection, expression of both ligands significantly increased (hMDM1: RFI: 2.01 ± 1.09 and hMDM2: RFI: 1.78 ± 0.41, respectively) (Figure 1A). Interestingly, basal surface expression of PD-L1 on hMDDC (RFI: 2.13 ± 0.37) was higher compared to hMDM1 or hMDM2, which however did not increase upon *Lm* infection (RFI: 2.33 ± 0.36) (Figure 1A). Focusing on PD-L2 expression, also a low basal surface expression was observed on hMDM1 (RFI: 1.91 ± 0.88), hMDM2 (RFI: 1.90 ± 0.74), and hMDDC (RFI: 3.27 ± 2.36), respectively. During *Lm* infection, PD-L2 surface expression significantly increased on hMDM1 (RFI: 2.78 ± 1.44) (Figure 1A). However, surface expression levels of PD-L2 on hMDM2 and hMDDC did not differ in presence or absence of *Lm* infection. Taken together, we show that PD-L1 and PD-L2 are expressed on all three host cell types and their expression is partially upregulated by *Lm* infection, which is a prerequisite to modulate the PD-1/PD-L axis by anti-PD-1 blockade.

### PHA Treatment Mimics T-Cell Exhaustion As Determined by Surface Expression of Various Marker Proteins

In the chronic LCMV mouse model, where T-cell exhaustion was initially defined, persistent antigen stimulation leads to T-cell exhaustion, which is characterized by a stepwise upregulation of several inhibitory molecules like PD-1, PD-L1, 2B4, lymphocyte-activation gene 3 (LAG-3), T-cell immunoglobulin and mucin-domain containing-3 (TIM-3), and CD160 (28, 29). To mimic an exhausted phenotype, we stimulated PBLs with PHA for 6 days. In contrast to CD3/CD28 stimulation, PHA stimulation has been reported to lead to an expansion of functionally impaired T-cells (30). After 6 days of PHA stimulation, we observed that most of the B-cells and NK-cells were overgrown by T-cells shown by the significant increase of CD3 positivity (96.3 ± 1.58%), compared to the unstimulated control (80.9 ± 5.59%) (Figure 1B). To assure that the T-cells were not functionally impaired by senescence, characterized by downregulation or loss of CD28, we additionally analyzed expression of this costimulatory molecule. CD28 expression tended to be higher in PHA-stimulated T-cells (RFI: 15.65 ± 10.06) compared to the untreated control (RFI: 12.7 ± 5.81) (Figure 1B). Therefore, we excluded T-cell senescence. Next, we investigated surface expression of several T-cell exhaustion markers on T-cells of PHA-prestimulated PBLs (PBLs^PHA^). Compared to the unstimulated control, PHA-prestimulated T-cells expressed the coinhibitory molecules PD-1 (RFI: 1.87 ± 0.55; 7.41 ± 2.50), PD-L1 (RFI: 4.09 ± 1.42; 7.52 ± 2.18), 2B4 (RFI: 4.27 ± 1.71; 9.70 ± 3.71), LAG-3 (RFI: 1.66 ± 0.34; 2.95 ± 0.35), and TIM-3 (RFI: 1.47 ± 0.16; 28.73 ± 18.65) to a significantly higher degree (Figure 1B). CD160 expression was not affected by PHA stimulation (RFI: 1.17 ± 0.07; 1.45 ± 0.56) (Figure 1B). Due to the high expression of several inhibitory molecules, we considered T-cells of PBLs^PHA^ to resemble exhausted T-cells. Thus, we subsequently used PBLs^PHA^ (containing PD-1^+^ T-cells) in autologous cocultures with the three *Lm* host cell types (PD-L1^+^ and PD-L2^+^) to investigate how T-cell effector functions are affected by nivolumab treatment.

### PD-1/PD-L Blockade-Induced T-Cell Proliferation and *Lm* Infection Rate Are Dependent on the Initial Host Cell Phenotype

**Figure 2:**
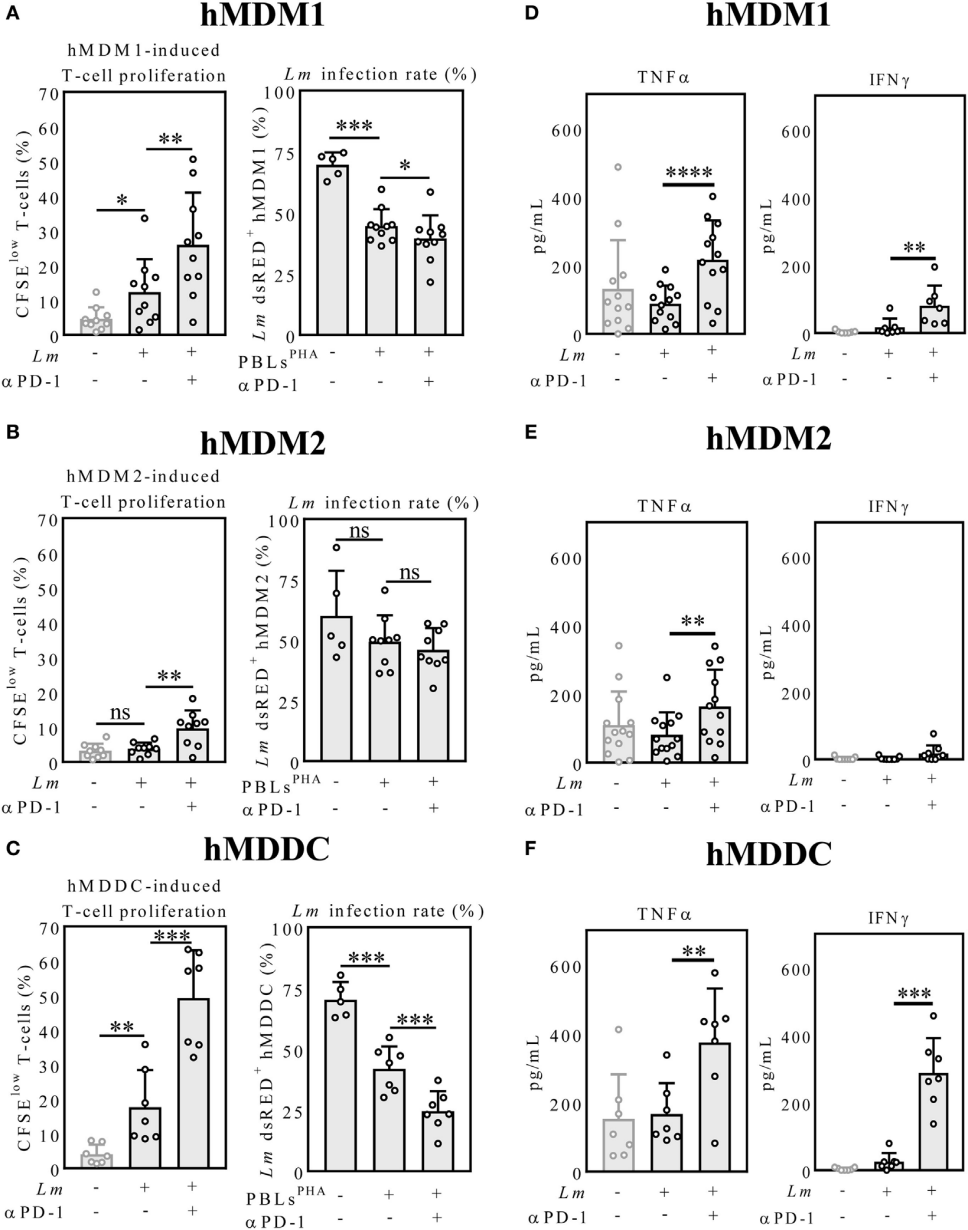
Host cell phenotype-dependent T-cell proliferation, parasite killing and cytokine release upon PD-1 blockade. **(A–C)** CFSE-labeled autologous PBLs^PHA^ were cocultivated with *Lm*-infected host cells and blocked with nivolumab (αPD-1). After 5 days, cells were harvested and analyzed by flow cytometry (hMDM/hMDDC gated *via* their FSC/SSC properties, T-cells additionally *via* CD3). Data are presented as mean percentage of CFSE^low^ proliferating T-cells ± SD or *Lm* dsRED-infected hMDDC ± SD, respectively. **(D–F)** IFNγ and TNFα were measured in the coculture supernatants by ELISA as described. Data are presented as mean pg/mL ± SD. For statistics, a parametric paired *t*-test was performed, *P* < 0.05 is considered statistically significant (*P < 0.05, **P < 0.01, ***P < 0.001, ****P < 0.0001). At least three independent experiments were performed (*N* = 5–13). hMDM1, proinflammatory human monocyte-derived macrophages type 1; hMDM2: anti-inflammatory human monocyte-derived macrophages type 2; hMDDC, human monocyte-derived dendritic cells; *Lm, Leishmania major*; PD-1, programmed death-1; PBL, peripheral blood lymphocytes; TNFα, tumor necrosis factor α; IFNγ, interferon-γ; CFSE, 5(6)-carboxyfluorescein diacetate N-succinimidyl ester.

First, we addressed, whether T-cell effector functions of PBLs^PHA^ can be reinvigorated by the therapeutic anti-PD-1 antibody nivolumab and whether this is influenced by the type of antigen presenting cell. Therefore, we compared hMDM1, hMDM2 and hMDDC, respectively, as *Lm* antigen presenting cells in cocultures with autologous PBLs^PHA^. Cocultivation of *Lm*-infected hMDM1 with PBLs^PHA^ significantly increased T-cell proliferation (12.32 ± 9.49%), compared to the uninfected control (4.63 ± 3.33%) (Figure 2A). This indicates that PHA-prestimulated T-cells have residual effector functions. Furthermore, by blocking PD-1, T-cell proliferation (25.96 ± 15.07%) was significantly enhanced (Figure 2A). Concomitantly, we observed a significant reduction in *Lm* infection in the sample cocultivated with PBL^PHA^ (44.62 ± 6.87%) compared to infected hMDM1 only (69.66% ± 5.07%), which consequently was reduced to a stronger extent upon PD-1 blockade (39.47 ± 9.48%) (Figure 2A). In cocultures of *Lm*-infected hMDM2 with autologous PBL^PHA^, no significant difference in T-cell proliferation was observed (3.84 ± 1.74%) compared to the uninfected control (3.21 ± 2.05%) (Figure 2B). However, PD-1 blockade enhanced T-cell proliferation significantly in the infected sample (9.71 ± 5.13%) (Figure 2B). Consistently, *Lm* infection rate of hMDM2 in the presence of PBLs^PHA^ was not decreased significantly (49.33 ± 10.90%) compared to infected hMDM2 only (63.49 ± 23.31%). Surprisingly, no significant effect on *Lm* infection could be detected when PD-1 was blocked (45.99 ± 8.94%), even though T-cell proliferation was increased (Figure 2B). Compared to hMDM1 and hMDM2 *Lm*-infected hMDDC induced the highest T-cell proliferation in the PBLs^PHA^ coculture (17.71 ± 10.73%) and, after PD-1 blockade, enhanced T-cell proliferation the most (49.30 ± 13.81%) (Figure 2C). Consequently, less *Lm*-infected hMDDCs were observed in the presence of PBLs^PHA^ (42.02 ± 9.17%) and PD-1 blockade dampened the infection rate of hMDDCs the most (24.68 ± 8.15%), compared to hMDM1 and hMDM2 (Figure 2C). PD-1 blockade in the absence of PBLs^PHA^ did not influence *Lm* infection rate in hMDM1, hMDM2 or hMDDC (Figure S1 in Supplementary Material). Furthermore, we showed that PD-1 is blocked on T-cells throughout the whole coculture experiment, indicating that the used amount of nivolumab is sufficient (Figure S2 in Supplementary Material). Our data demonstrate that T-cell function is enhanced by PD-1 blockade, however, the extent of the effects strongly depends on the *Lm* host cell phenotype.

### TNFα and IFNγ Release Is Strongly Enhanced after PD-1 Blockade in the *Lm*-Infected hMDDC Coculture

The proinflammatory cytokines TNFα and IFNγ play a critical role in shaping the immune response against *Leishmania* infection (31). Thus, we analyzed TNFα and IFNγ levels in the coculture supernatants by ELISA. PD-1 blockade significantly increased TNFα levels in the infected hMDM1 samples (216.60 ± 114.90 pg/mL; 88.57 ± 52.52 pg/mL) (Figure 2D). Also IFNγ release was augmented by PD-1 blockade in the infected hMDM1 samples (80.61 ± 59.73 pg/mL; 16.95 ± 25.68 pg/mL) (Figure 2D). TNFα levels in the hMDM2 samples were similar to the hMDM1 samples. In contrast to hMDM1, IFNγ was barely detectable in the PD-1-blocked infected hMDM2 sample (17.62 ± 24.18 pg/mL) (Figure 2E). Regarding *Lm*-infected hMDDC, PD-1 blockade significantly increased TNFα levels in the supernatants (374.50 ± 156.60 pg/mL; 167.20 ± 89.77 pg/mL). IFNγ levels in the supernatant of *Lm*-infected hMDDC were low (25.58 ± 25.54 pg/mL), but highly enhanced by PD-1 blockade (288.40 ± 103.20 pg/mL) (Figure 2F). This suggests that effects induced by PD-1 blockade may be mediated by TNFα and IFNγ. To investigate this idea, we performed a correlative analysis and neutralized both cytokines.

### TNFα but Not IFNγ Neutralization Partially Reversed Effects of Nivolumab Treatment

**Figure 3:**
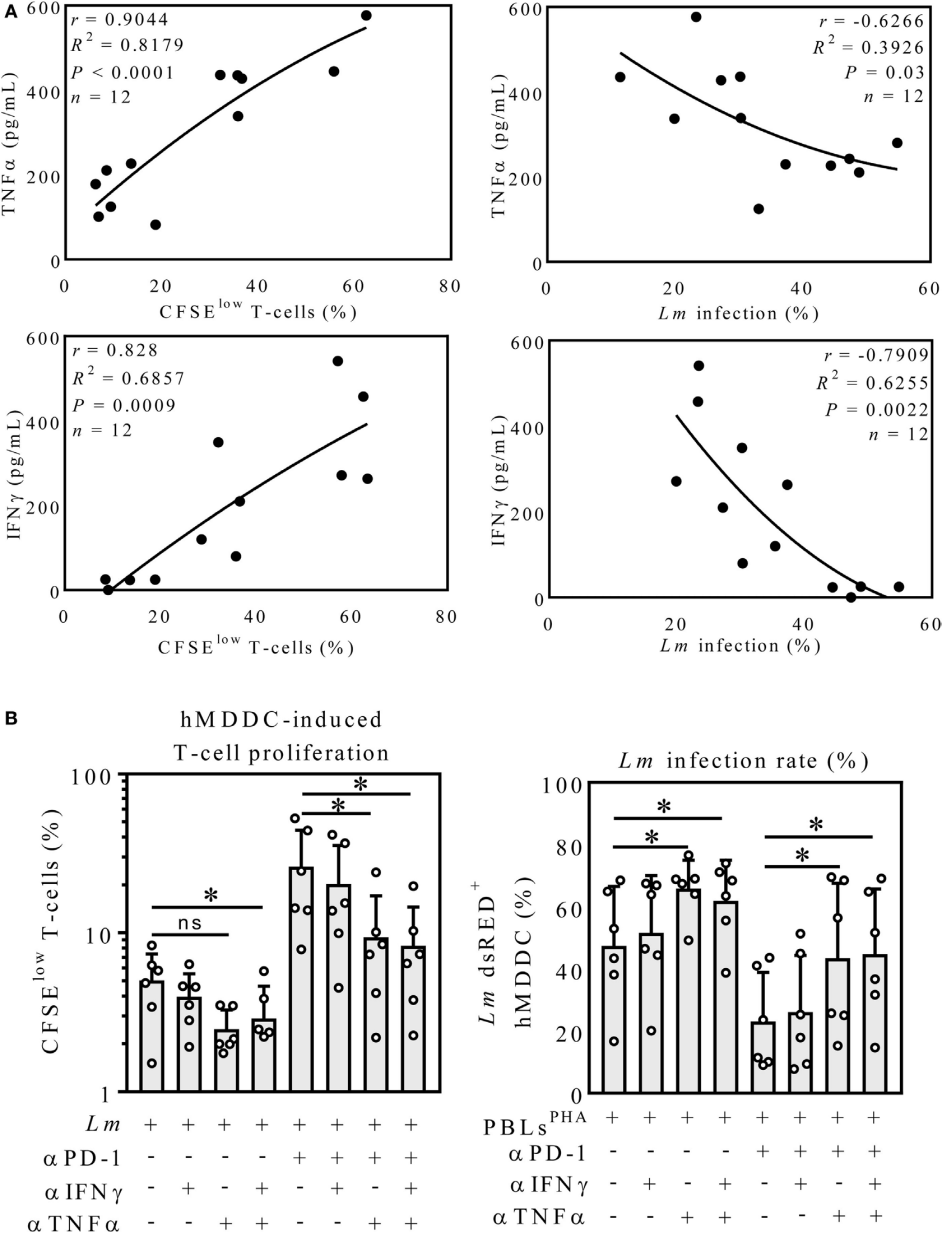
Neutralization of TNFα but not IFNγ partially reversed PD-1 blockade-mediated effects. **(A)** TNFα- and IFNγ-release correlates with T-cell proliferation. All *Lm*-infected hMDDC samples cocultured with PBL^PHA^ in presence or absence of anti-PD-1 (αPD-1) were considered (TNFα and IFNγ: *n* = 12) and Pearson correlation coefficients (*r*), the coefficient of determination (*R^2^*) plus the significance level (*P*) were calculated for the indicated datasets. *r* ≥ 0.7 = positive correlation; *r* ≤ −0.7 = inverse correlation. **(B)** CFSE-labeled autologous PBLs^PHA^ were cocultivated with *Lm*-infected hMDDC, nivolumab (αPD-1), IFNγ neutralizing antibody (αIFNγ) and TNFα neutralizing antibody (αTNFα) as indicated. After 5 days, cells were harvested and analyzed by flow cytometry (hMDM/hMDDC gated *via* their FSC/SSC properties, T-cells additionally *via* CD3). Data are presented as mean percentage of CFSE^low^ proliferating T-cells ± SD or *Lm* dsRED-infected hMDDC ± SD, respectively. For statistics, a parametric paired *t*-test was performed, *P* < 0.05 is considered statistically significant, *P < 0.05, **P < 0.01. At least three independent experiments were performed (*N* = 5–6). hMDDC, human monocyte-derived dendritic cells; *Lm, Leishmania major*; PD-1, programmed death-1; PBL, peripheral blood lymphocytes; TNFα, tumor necrosis factor α; IFNγ, interferon-γ; CFSE, 5(6)-carboxyfluorescein diacetate N-succinimidyl ester.

To assess whether cytokine release correlates with T-cell proliferation or *Lm* infection rate, we performed a correlative analysis. To this end, we focused on *Lm*-infected hMDDC, because these host cells displayed the strongest effects mediated by PD-1 blockade in the coculture experiment (Figures 2C,F). We only used the datasets of infected samples in presence and absence of nivolumab (Figure 3A). TNFα release correlated with T-cell proliferation (*r* = 0.90). TNFα release tendentially correlated inversely with infection rate (*r* = −0.63). There was a positive correlation between IFNγ release and T-cell proliferation (*r* = 0.83). Also infection rate tendentially correlated inversely with IFNγ release (*r* = −0.79). To investigate a causal connection between increased TNFα/IFNγ release and T-cell proliferation/parasite survival, we neutralized TNFα and IFNγ. Neutralization of IFNγ in the infected sample had no significant effect on T-cell proliferation (Figure 3B). Neutralization of TNFα tendentially reduced T-cell proliferation compared to *Lm* infection only (2.48 ± 0.80%; 5.02 ± 2.36%). Simultaneous neutralization of both cytokines reduced T-cell proliferation significantly (2.89 ± 1.74%) compared to the untreated control. PD-1 blockade increased T-cell proliferation (26.18 ± 18.11%). Neutralization of IFNγ did not decrease PD-1 blockade-induced T-cell proliferation (20.29 ± 15.08%), whereas anti-TNFα treatment had a significant reducing effect (9.37 ± 7.72%). Simultaneous neutralization of both cytokines did not enhance this effect (8.30 ± 6.25%). In the absence of anti-PD-1 antibody, *Lm* infection rate (47.42 ± 19.11%) was not increased in the IFNγ neutralized sample (51.55 ± 18.48%). Interestingly, TNFα neutralization highly increased parasite survival (65.85 ± 9.31%), which was not further enhanced by additional neutralization of IFNγ (61.87 ± 13.05%). Infection rate in hMDDCs decreased in the presence of anti-PD-1 (23.12 ± 15.78%). Neutralizing single or both cytokines revealed the same effect, as observed for the samples without nivolumab. Again, only TNFα neutralization significantly increased parasite survival (43.40 ± 24.17%) (Figure 3B). Blocking with an isotype control did not alter proliferation or parasite survival (data not shown). Even though the correlative analysis pointed toward a role for IFNγ in T-cell proliferation and parasite killing, only neutralization of TNFα partially reversed PD-1 blockade-mediated effects. On the other hand, TNFα neutralization already improved parasite survival in the absence of PD-1 blockade, indicating that TNFα might act independently of PD-1.

### PD-1 Blockade Shifts *Lm*-Induced CD4^+^-T-Cells toward a T_H_1- or T_H_1/T_H_2 Phenotype

**Figure 4:**
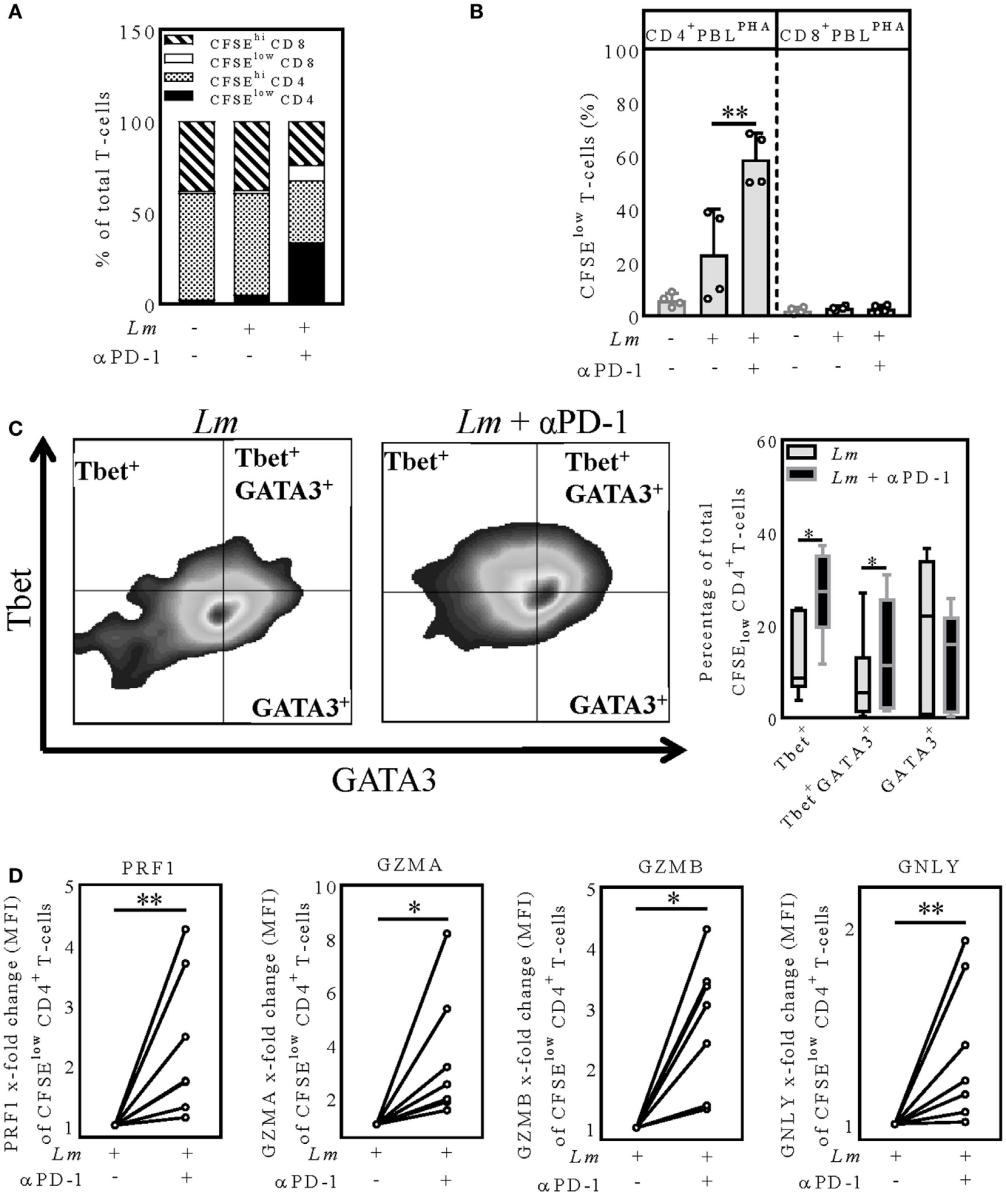
PD-1 blockade shifts proliferating CD4^+^-T-cells toward a T_H_1 or T_H_1/T_H_2 phenotype and increases expression of cytolytic effector molecules **(A,C,D)** CFSE-labeled autologous PBLs^PHA^ were cocultivated with *Lm*-infected hMDDC (MOI = 10) and nivolumab (αPD-1). After 5 days, cells were harvested, immunostained **(A)** (CD3, CD4, CD8) **(C,D)** (CD3, CD4, PRF1, GZMA, GZMB, GNLY, Tbet, GATA3) and analyzed by flow cytometry. T-cells were gated *via* their FSC/SSC properties and the labeled antibodies. Data are presented as **(A)** percentage of total T-cells, **(B,C)** mean ± SD percentage or **(D)** mean ± SD *x*-fold change (MFI) relative to the untreated infected sample. At least two independent experiments were performed (*N* = 4–8). **(B)** CFSE-labeled autologous CD4^+^ or CD8^+^ PBLs^PHA^ were cocultivated with *Lm*-infected hMDDC and nivolumab (αPD-1). After 5 days, cells were harvested and analyzed by flow cytometry. T-cells were gated *via* their FSC/SSC properties and CD3. At least two independent experiments were performed (*N* = 4). Statistics were calculated **(B)** using the parametric paired *t*-test or **(C,D)** the Wilcoxon matched-pairs signed rank test; *P* < 0.05 is considered statistically significant (*P < 0.05; **P < 0.01). hMDDC, human monocyte-derived dendritic cells; *Lm, Leishmania major*; PHA, phytohemagglutinin; PBL, peripheral blood lymphocytes; PRF1, perforin; GZMA, granzyme A; GZMB, granzyme B; GNLY, granulysin; Tbet, T-box transcription factor; GATA3, GATA-binding protein 3; CFSE, 5(6)-carboxyfluorescein diacetate N-succinimidyl ester; MFI, mean fluorescence intensity.

We sought to examine the T-cell response after PD-1 blockade in more detail. Therefore, we characterized the proliferating CFSE^low^ and the non-proliferating CFSE^hi^ CD4^+^ and CD8^+^-T-cells in the hMDDC coculture (Figure 4A). T-cells were composed of ~60% CD4^+^ cells and ~40% CD8^+^ (uninfected control); in the PD-1 blocked *Lm*-infected sample almost exclusively CD4^+^-T-cells proliferated (33.89 ± 17.95% CFSE^low^ CD4^+^; 8.52 ± 5.73% CFSE^low^ CD8^+^) (Figure 4A). We confirmed this finding in a coculture experiment using purified CD4^+^ PBLs^PHA^ or CD8^+^ PBLs^PHA^ and detected T-cell proliferation only in the presence of CD4^+^ PBLs^PHA^ (Figure 4B). Hence, PD-1 blockade mainly enhanced effector functions of CD4^+^ T-cells. As mentioned earlier, in the classical mouse model of leishmaniasis the induction of a T_H_1 response leads to healing, whereas induction of T_H_2 cells promotes disease (2). In human leishmaniasis such a T_H_1/T_H_2 dichotomy is not that evident (5, 6). Thus, we examined in our model whether the T_H_1-specific transcription-factor Tbet and the T_H_2-specific transcription-factor GATA3 are differentially expressed upon PD-1 blockade. The representative dot-blots on the left of Figure 4C indicate differential intra-nuclear expression of Tbet and GATA3 in presence or absence of nivolumab in the *Lm*-infected sample. Compared to the untreated infected sample, a significantly higher number of Tbet^+^ (13.06 ± 8.35%; 26.06 ± 9.46%) and Tbet^+^/GATA3^+^ (8.68 ± 9.22%; 14.41 ± 11.33%) proliferating CD4^+^ T-cells was measured in the presence of nivolumab, whereas the percentage of GATA3^+^ T-cells was tendentially lower (18.77 ± 16.68; 12.68 ± 10.54) (Figure 4C). We conclude that PD-1 blockade shifts *Lm*-induced CD4^+^ T-cells more toward a T_H_1- or T_H_1/T_H_2 phenotype. The higher percentage of T_H_1 T-cells might be implicated in the improved parasite killing.

### Intracellular Expression of Perforin, Granzymes, and Granulysin (GNLY) Is Increased in PD-1-Blocked *Lm*-Induced CD4^+^ T-Cells

CD4^+^ T-cells can release an extensive repertoire of cytolytic effector molecules, used either to help or to initiate apoptosis of a target cell (32). To examine, whether PD-1 blockade increases expression of such cytolytic effector molecules, we analyzed intracellular expression of perforin (PRF1), granulysin (GNLY), granzyme A (GZMA), and granzyme B (GZMB) in CFSE^low^CD4^+^-T-cells in the hMDDC coculture using flow cytometry (Figure 4D). Intracellular expression of PRF1 was significantly higher (1.13–4.26-fold increase) in the proliferating CD4^+^-T-cells after PD-1 blockade relative to the untreated infected sample (Figure 4D). Like for PRF1, we measured a higher GZMA (1.53–8.17-fold increase), GZMB (1.31–4.3-fold increase) and GNLY expression (1.01–1.93-fold increase) in the infected samples when PD-1 was blocked (Figure 4D). To conclude, we determined intracellular PRF1, GNLY, GZMA, and GZMB to be increased in the *Lm*-induced proliferating CD4^+^ T-cells upon PD-1 blockade. This strongly suggests that reduced parasite survival upon PD-1 blockade is mediated in part by PRF1, GZMA, GZMB, and GNLY.

## Discussion

The function of the coinhibitory PD-1/PD-L pathway and the phenomenon of T-cell exhaustion have been well defined in the chronic LCMV mouse model (18, 28, 33). Moreover, T-cell exhaustion was demonstrated to occur in many human diseases caused by viruses (34, 35), bacteria (36), fungi (37), cancer (38, 39) and protozoan infections including leishmaniasis (24, 40, 41). Remarkably, effector functions of exhausted T-cells can be reinvigorated by blocking the PD-1/PD-L interaction, thereby reducing LCMV loads in the chronic LCMV mouse model (18). This principle has been extended to other applications, like human cancers (42) and animal infection models (21, 23). Experimental animal models of leishmaniasis indicate that the use of PD-1/PD-L blocking antibodies might be beneficial for treatment of chronic forms of leishmaniasis (21–23).

Previously we reported that *Lm-*infected macrophages generated from monocytes of *Leishmania*-naive German blood donors induce a MHC class II-dependent T-cell response in autologous *in vitro* cocultures. This early T-cell response reduced parasite load in the infected macrophages (11, 27). PD-1/PD-L interactions are not prominent during the early T-cell priming phase, but they regulate the T-cell response during the effector phase (43). During chronic infections and cancer, PD-1/PD-L interactions play an important role in induction of T-cell exhaustion (44).

To investigate PD-1/PD-L interactions in *Lm* infection of human myeloid and lymphoid cells, we used a newly established *in vitro* model. In this model, we mimicked exhausted T-cells by stimulating PBLs with PHA prior to cocultivation with autologous *Lm*-infected host cells. PHA is a mitogen used for polyclonal activation and expansion of T-cells (45). Furthermore, it is reported to lead to functionally impaired T-lymphocytes in contrast to CD3/CD28 stimulation (30). We confirmed PHA-stimulated T-cells express high levels of PD-1, PD-L1, LAG-3, TIM-3, and 2B4, and thus resemble exhausted T-cells.

In the current study, we found the anti-PD-1 antibody nivolumab to enhance T-cell effector functions and reduce parasite survival differently depending on the *Lm* host cell phenotype that was initially infected. Our data suggest *Lm*-infected hMDM2 not to be target of PD-1/PD-L-mediated inhibition, whereas PD-1/PD-L interactions strongly inhibit T-cell effector functions on *Lm*-infected hMDDC. A reason for this might be that both PD-1 ligands are higher expressed on hMDDC compared to hMDM2. Furthermore, antigen-presenting dendritic cells are much more potent in inducing T-cell responses compared to macrophages. Arnold et al. compared differently polarized monocyte-derived macrophages and dendritic cells in their ability to induce autologous T-cells by using mycobacterial purified protein derivate as recall antigen or keyhole limpet hemocyanin as a primary antigen for naive T-cell responses. In this context dendritic cells (hMDDC) induced the highest levels of T-cell proliferation to both antigens, whereas LPS + IFNγ-treated macrophages (comparable to hMDM1) were less effective in inducing antigen-specific T-cell responses followed by IL-4-treated macrophages (comparable to hMDM2) (46).

Focusing on hMDDC, we could partially reverse effects of PD-1 blockade by neutralization of soluble TNFα. Chiku et al. demonstrated elevated levels of TNFα and a concomitant decrease of parasite load after PD-1/PD-L blockade in the canine model of visceral leishmaniasis (47).

Furthermore, we found that neutralization of IFNγ, which was induced after PD-1 blockade in the hMDDC samples, had no significant effect on T-cell proliferation or parasite load. IFNγ is reported to mediate resistance to *Lm* in mice by inducing iNOS expression (2). In human myeloid cells iNOS (or NOS2) expression and function is controversially debated (48, 49). *In vitro* generated human myeloid cells are unable to express functional iNOS (NOS2) because the essential iNOS cofactor BH4 is missing (48). In contrast to mice, ROS play an important role in parasite control during human Leishmaniasis. IFNγ was shown to induce ROS in *Leishmania*-infected human monocytes, which dampened overall parasite load (8). Furthermore, ROS-dependent parasite control was rather evident in monocytes from CL patients than in monocytes from healthy individuals (7). Thus, it could be that IFNγ-induced ROS production in *Lm*-infected hMDDC was to low to see significant effects of IFNγ neutralization on infection rate in our *in vitro* model. One reason for why we did not observe significant differences in T-cell proliferation might be the general increased survival of T-cells upon IFNγ. However, IFNγ does not increase the number of antigen-specific T-cell divisions. This was shown, e.g., for murine OVA-specific CD4^+^-T-cells (50).

In our *in vitro* model, preferentially CD4^+^-T-cells proliferated upon *Lm* infection and nivolumab treatment. In the classical mouse model of *Lm*, induction of T_H_1 response leads to resistance whereas the induction of a T_H_2 response promotes disease (2). Human cutaneous leishmaniasis patients with moderate disease symptoms show a balanced T_H_1/T_H_2 response, whereas an imbalance of T_H_1/T_H_2 is associated with disease severity (51). Experiments with blood of prostate and advanced melanoma cancer patients revealed that PD-1 blockade augments T_H_1 responses und suppresses T_H_2 responses (52). By analyzing intranuclear expression of the T_H_1-specific Tbet and T_H_2-specific GATA3, we found PD-1 blockade to shift *Lm*-induced CD4^+^ T-cells more toward a T_H_1 or T_H_1/T_H_2 phenotype, respectively. Thus, the higher abundance of T_H_1 T-cells and their effector functions may be implicated in reduction of *Lm* infection.

Specific killing of intracellular parasites in a concerted action of perforin, granzymes, and granulysin (expressed by T-cells) with minimal collateral damage to the host cell was demonstrated in transgenic mouse models (53). PD-1 blockade is described to increase perforin, granzyme B, and granulysin expression in T-cells of tuberculosis and cancer patients (54). Focusing on *Lm*-induced CD4^+^ T-cells, we detected increased intracellular levels of perforin, granzyme A and B, and granulysin after PD-1 blockade. This suggests that cytolytic molecules might contribute to the reduction of *Lm* in hMDDC.

Altogether, our data suggest how the PD-1/PD-L axis could modulate *Lm* host cells and CD4^+^ T-cells in patients suffering from chronic forms of leishmaniasis. So far, the focus of research groups is mostly on CD8^+^ T-cell exhaustion, which was observed in diffuse cutaneous (40) and visceral human leishmaniasis (24). In case of the cutaneous forms of human leishmaniasis, CD8^+^ T-cells have a dual role (55) but their contribution in resolving primary cutaneous *Leishmania* infection might be negligible (56, 57). CD4^+^ T-cells activate leishmanicidal functions of infected macrophages and dendritic cells. In our experiments infected dendritic cells benefit the most from PD-1 blockade, as this strongly enhanced CD4^+^ T-cell effector functions and parasite killing. Additionally we found increased levels of maturation markers on *Lm*-infected dendritic cells after PD-1 blockade (Figure S3 in Supplementary Material). To induce a strong cell-mediated immunity, e.g., after vaccination, adequate maturation of dendritic cells is important. In animal models, it was demonstrated that vaccination using *L. mexicana* LPG induced PD-1/PD-L2 expression on several immune cells in a dose dependent fashion (58). Blocking the PD-1/PD-L interaction could be a valuable approach to enhance efficacy of *Leishmania* vaccine candidates. Dendritic cell-based immunotherapy in combination with antimonials has been shown to significantly reduce parasite burden in experimental models of visceral leishmaniasis (59, 60). This approach might also benefit from PD-1 checkpoint inhibitors.

Collectively, by using a limited reductionist approach the present work provides new insights regarding the PD-1/PD-L axis in *Lm* infection of primary human cells and its consequence for adaptive immunity. Further experiments using material obtained from chronic leishmaniasis patients can contribute to a better understanding of PD-1 blockade-mediated effects.

## Author Contributions

CF, PC, ZW, GR, and GVZ contributed to conception and design of the study; CF, KA, and GANN performed and analyzed experiments; CF wrote the first draft of the manuscript; all authors contributed to manuscript revision, read, and approved the submitted version.

## Conflict of Interest Statement

The authors declare that the research was conducted in the absence of any commercial or financial relationships that could be construed as a potential conflict of interest.
