# Supplementary material for: Nivolumab Enhances In Vitro Effector Functions of PD-1+ T-Lymphocytes and Leishmania-Infected Human Myeloid Cells in a Host Cell-Dependent Manner
Source: Front Immunol. 2017 Dec 22;8:1880. doi: 10.3389/fimmu.2017.01880 (PMC5743744; doi:10.3389/fimmu.2017.01880)
Supplement: Supplementary file 1 [file Data_Sheet_1.docx]

Supplementary Material

Nivolumab enhances *in vitro* effector functions of PD-1^+^ T-lymphocytes and *Leishmania*-infected human myeloid cells in a host cell-dependent manner

Christodoulos Filippis, Katharina Arens, Gaetan Aime Noubissi Nzeteu, Gabriele Reichmann, Zoe Waibler, Peter Crauwels and Ger van Zandbergen*

***Correspondence:** Corresponding Author: Ger.Zandbergen@pei.de

Supplemental Table 1: List of primary labeled antibodies

| **Antibody** | **Clone** | **Manufacturer** |
| --- | --- | --- |
| mouse anti-human PD-L1 PE | MIH1 | BD Biosciences |
| mouse anti-human PD-L2 PE | 24F.10C12 | Biolegend® |
| mouse anti-human PD-1 PE | MIH4 | BD Biosciences |
| mouse anti-human 2B4 APC | C1.7 | Biolegend® |
| mouse anti-human LAG-3 PE | T47-530 | BD Biosciences |
| mouse anti-human TIM-3 APC | F38-2E2 | Biolegend® |
| mouse anti-human CD160 PE | BY55 | Biolegend® |
| mouse anti-human CD28 PE | CD28.2 | BD Biosciences |
| mouse anti-human CD3 Pacific Blue™ or APC | UCHT1 | Biolegend® |
| mouse anti-human CD4 PE | RPA-T4 | BD Biosciences |
| mouse anti-human CD8 Pacific Blue™ | RPA-T8 | BD Biosciences |
| mouse anti-human CD80 V450 | L307.4 | BD Biosciences |
| mouse anti-human CD83 APC | HB15e | BD Biosciences |
| mouse anti-human CD86 FITC | 2331 (FUN-1) | BD Biosciences |
| mouse anti-human CD40 PE | 5C3 | BD Biosciences |
| mouse anti-human Perforin APC | dG9 | Biolegend® |
| mouse anti-human Granulysin Alexa Fluor® 647 | DH2 | Biolegend® |
| mouse anti-human Granzyme A Alexa Fluor® 647 | CB9 | Biolegend® |
| mouse anti-human Granzyme B Alexa Fluor® 647 | GB11 | Biolegend® |
| mouse anti-human Tbet Pacific Blue™ | 4B10 | Biolegend® |
| mouse anti-human GATA3 Alexa Fluor® 647 | L50-823 | BD Biosciences |

Supplemental Figure 1: PD-1-blockade does not impact *Lm* infection rate in absence of PBL^PHA^. hMDM1, hMDM2 or hMDDC were incubated with/without *Lm* dsRED (MOI 10) for 24h. After washing extracellular parasites away, infected cells were further incubated in a 96-U-well plate (final concentration: 0,125 * 10^6^ cells/ml) together with nivolumab antibody (final concentration: 0.625 µg/ml). After 5d, cells were harvested and *Lm* infection rate was analyzed by flow cytometry (Gating via FSC/SSC properties and *Lm* dsRED). Two independent experiments were performed (N = 5). Data is presented as mean ± SD. Statistics were calculated using a parametric paired t-test, *P* < 0.05 is considered statistically significant). hMDM1, pro-inflammatory human monocyte-derived macrophages Type 1; hMDM2, anti-inflammatory human monocyte-derived macrophages Type 2; hMDDC, human monocyte-derived dendritic cells; *Lm*, *Leishmania major*; PD-1, programmed death-1.

Supplemental Figure 2: PD-1 receptor is still blocked after 5d co-culture. hMDM1, hMDM2 or hMDDC were incubated with *Lm* (MOI 10) for 24h. After washing extracellular parasites away, infected cells were cocultivated with autologous PBL^PHA^ and nivolumab (αPD-1 block). After 5d, cells were harvested and T-cells analyzed by flow cytometry (Gating via FSC/SSC properties + CD3). PD-1 was detected using human IgG4 nivolumab (αPD-1 detect) plus an anti-human IgG PE-labeled Fab_2_ fragment (αhuIgG R-PE). The Fab_2_ fragment alone did detect nivolumab (αPD-1 block) that was added at the beginning of the co-culture experiment. Adding fresh nivolumab (αPD-1 detect) did not increase PD-1 positivity in the PD-1-blocked samples (αPD-1 block). Two independent experiments were performed (N = 3-4). Data is presented as mean ± SD. hMDM1, pro-inflammatory human monocyte-derived macrophages Type 1; hMDM2, anti-inflammatory human monocyte-derived macrophages Type 2; hMDDC, human monocyte-derived dendritic cells; *Lm*, *Leishmania major*; PD-1, programmed death-1.

Supplemental Figure 3: PD-1 blockade tendentially increased expression of maturation markers on hMDDC. hMDDC were incubated with *Lm* (MOI 10) for 24h. After washing extracellular parasites away, infected cells were cocultivated with autologous PBL^PHA^ and nivolumab (αPD-1). After 5d cells were harvested, immunostained (CD80, CD83, CD86 and CD40) and analyzed by flow cytometry. hMDDCs were first gated via their FSC/SSC properties and second by the indicated marker. Data is presented as mean ± SD RFI (ratio of the mean fluorescence intensity of specific markers to the mean fluorescence intensity of isotype controls). Statistics were calculated by Wilcoxon matched-pairs signed rank test; *P* < 0.05 is considered statistically significant (**P* < 0.05). At least two independent experiments were performed (n = 3-6). hMDDC, human monocyte-derived dendritic cells; *Lm*, *Leishmania major*; PD-1, programmed death-1.
